# In Vivo and In Vitro Studies Assessing the Safety of Monosodium Glutamate

**DOI:** 10.3390/foods13233981

**Published:** 2024-12-09

**Authors:** Tania Merinas-Amo, Rocío Merinas-Amo, Ángeles Alonso-Moraga, Rafael Font, Mercedes Del Río Celestino

**Affiliations:** 1 Department of Genetics, University of Córdoba, 14071 Córdoba, Spain; tania.meram@gmail.com (T.M.-A.); rocio.merinas@gmail.com (R.M.-A.); ge1almoa@uco.es (Á.A.-M.); 2 Agri-Food Laboratory, CAGPDS, Av. Menéndez Pidal, s/n, 14080 Córdoba, Spain; rafaelm.font@juntadeandalucia.es

**Keywords:** antitoxicity, cytotoxicity, DNA damage, *Drosophila melanogaster*, food additive, leukaemia cells, longevity, methylation status, monosodium glutamate, toxicity

## Abstract

The controversial results of research on monosodium glutamate demand a new data corpus for the overall safety evaluation. Both animal and cellular model systems have been used to add a multilevel scope on its biological effects. The *Drosophila melanogaster* animal model has been used to test a wide range of concentrations for safety purposes: toxicity, genotoxicity, longevity and health span. Medium concentrations corresponding to the human acceptable daily intake (ADI) (0.06 mg/mL) were not toxic nor genotoxic for *Drosophila* and safe for the lifespan parameters. Once safety was determined, the possible nutraceutical effects of monosodium glutamate was monitored in terms of antitoxicity, antigenotoxicity assays and health span. The results for protective activity against hydrogen peroxide were positive in terms of the medium concentration, antitoxic and antigenotoxic in terms of inhibiting the genotoxicity induced by the oxidative toxin up to 43.7% and increasing the health span expectancy by 32% in terms of days. Monosodium glutamate has been demonstrated to be cytotoxic against the model tumour cell line HL-60, not only in a necrotic way but through internucleosomal DNA fragmentation antitumour activity. The significant LINE1 DNA sequence methylation of HL-60 tumour cells induced by monosodium glutamate is a molecular marker for chemoprevention. Conclusions: the slight or non-significant positive nutraceutical and chemo preventive potential showed by monosodium glutamate at its ADI concentration can be considered as a safe dose for a moderate consumption.

## 1. Introduction

The way in which humans eat has dramatically changed from Palaeolithic times to the present day. Although this big leap corresponds to plant and animal domestication and subsequent breeding, we currently attend to a new event called food processing. In addition, to the discovery of new raw products suitable for human consumption, food technology is centred on either safety purposes or searching for new flavours. A wide variety of substances are added to food for increasing/lasting the quality and the sensorial appeal of the final processed product [1]. Colourings, preservatives, antioxidants, stabilisers, emulsifiers, thickeners, acidulants, acidity regulators, anticaking agents, flavour enhancers and sweeteners are being added to foods on a massive scale [2,3]. 

A glutamic acid salt, monosodium glutamate (commonly named as glutamate) (E-621), is a non-essential amino acid and the main excitatory neurotransmitter in the brain. It is involved in the improvement of palate perception [4]. Moreover, monosodium glutamate plays a key role in nitrogen homeostasis, and organisms do all they can to limit the bioavailability of monosodium glutamate, which can be neurotoxic in excess. As monosodium glutamate is never eaten alone, its bioavailability will be limited if not negligible, and no adverse effects are to be expected in adult humans [5]. Monosodium glutamate is added during food processing as a flavour enhancer and as a preservative. Formerly used very much in Asian cooking, it is now used worldwide. Databases of the different studies contain controversial results related to its dosage and metabolic pathways. 

Monosodium glutamate improves the palatability and acceptability of hospital and school foods [6,7] with low fat and salt content, so it can be useful to reduce sodium intake without reducing the sensory quality [8]. Nevertheless, besides this positive effect of monosodium glutamate use, many problems related to monosodium glutamate consumption related to idiopathic urticaria, metabolic disorders, obesity, heart and neurodegenerative diseases have been described [9,10,11]. The acceptable daily intake (ADI)information is also controversial, as different countries and international food organisations propose different values. Sasaki et al. [12] proposed 30 mg/kg/day body weight, which is coincident with the Joint FAO/WHO Expert Committee on Food Additives [13]; contrarily, the European Food Safety Authority [14] allows using 10 g/kg of food. 

Studies have demonstrated that monosodium glutamate has harmful side effects, notably in animals, such as the development of obesity and diabetes as well as hepatotoxic, neurotoxic and genotoxic consequences [15]. Moreover, studies also have related the consumption of monosodium glutamate with metabolic disorders, carcinogenesis and diseases [16,17,18,19]. JECFA examined acute, sub-chronic and chronic toxicity studies in rats, mice and dogs, together with studies on reproductive toxicity and teratology. Glutamate was found to have a very low acute oral toxicity [15].

The meta-analysis carried out by Gottardo et al. [20] yielded 42% of articles with positive 46% negatives and 12% neutral results. With respect to the clinical trials research included in this analysis, the most important limitation observed was related to the application of monosodium glutamate in animals, because most studies that showed a negative correlation with health were carried out using injectable methods. Thus, new molecular, cellular and individual targets are now possible for study using reliable methodologies, to precisely evaluate the biological effects of such as additives. 

According to Chalitchagorn et al. [21]; Ehrlich et al. [22]; Roman-Gomez et al. [23]; and Schmid [24], in general, all the substances act as preventive substances, blocking repetitive sequences of tumoural cells as theychange the usual hypomethylation status of these elements in cancer cells to a methylated status. With this aim, different studies have been carried out using diet components as possible modulator agents of DNA methylation in cancer cells. Moreover, epigenetic therapy against harmful effects of diet components could be a potential tool in chemotherapy because epigenetic alterations are reversible in contrast to genetic defects [25]. Dietary components could act in a dual way by increasing or decreasing the methylation levels depending on the specific gene promoter or the genomic region.

An excess of anything is harmful; monosodium glutamate utilization up to certain level does not have any adverse effects because glutamate is a nutritional amino acid. Therefore, more detailed and vigorous studies are required to check the underlying cause of drastic health effects (cardiac, circulatory, gastrointestinal, muscular and neurological disorders are some of the common examples) [26].

Considering the controversial information available on the various food additives, our main objective was to evaluate in a cross-sectional manner the biological effects of monosodium glutamate on degenerative processes by providing a new body of scientific data. To this end, an integrative study of biological activity at the individual, cellular and molecular levels was carried out based on in vivo and in vitro assays using two model systems: *Drosophila melanogaster*, as a holometabolous higher eukaryote, and the human tumour cell line HL-60.

*Drosophila melanogaster* is an excellent model organism for the investigation of degenerative diseases such as ageing, since it has a relatively short life expectancy; large numbers of individuals can be maintained in controlled laboratory conditions, and adults appear to show many of the manifestations of disease and cellular senescence observed in mammals [27]. For these reasons, flies have often been used to study physiological and pathological processes affecting lifespan, as well as to understand the relationship between nutrient metabolism and mechanisms of ageing [28]. This species is considered a model system, as it is a multicellular organism in which many aspects of gene expression parallel those of humans, such as cancer and ageing [29,30,31]. It is also known that more than 70% of genes related to different human degenerative diseases are present in this model organism [32], such as Parkinson’s and Alzheimer’s diseases and allergic diseases, among others; this add value for the extrapolation of the results obtained for *Drosophila* in the different studies of toxicity, antitoxicity, genotoxicity, antigenotoxicity and longevity, and their possible a priori effects on humans.

The HL-60 cell line is a lineage derived from a 36-year-old woman with acute promyelocytic leukaemia [33], consisting of myeloblastic or promyelocytic cells. The use of in vitro models of human transformed cells such as HL-60 has made important contributions to the disciplines of cancer, haematology and immunology, allowing the study of the effects of candidate compounds on cell growth inhibition, biological regulation of haematopoietic development or lymphokines [34], DNA damage (single and/or double DNA strand breaks associated with cell apoptosis or necrosis), as well as the modulation of methylation status by external agents [35] or the assessment of pneumococcal vaccine-induced anti-capsular antibodies by the standardised OPKA (opsonophagocytic killing) assay [36].

The final objectives of this research are as follows:

- To analyse the protective role of monosodium glutamate against oxidative genotoxin in the maintenance of DNA integrity using the in vivo model of *Drosophila melanogaster*.
- To study the effects of monosodium glutamate on survival in *Drosophila melanogaster*.
- To study in vitro the chemo preventive value of this food additive on human leukaemia HL-60 cells by means of cytotoxicity, fragmentation and DNA single- and double-strand breakage assays.
- To study the modification that monosodium glutamate induces in the genomic methylation pattern of HL-60 tumour cells.

## 2. Materials and Methods

### 2.1. Preparation of Sample

Considering its greatest impact on consumers and its high consumption and abundance in the diet, the food additive E-621 monosodium glutamate (Sigma-Aldrich, Darmstadt, Germany) was selected [37,38]. Six different concentrations of that flavour enhancer were tested. The range of concentration was calculated to make it comparable with the ADI in humans (120 mg/kg) [14] and considering the average daily intake (1 mg/day) and the average body weight of *D. melanogaster* (1 mg) [39]. A total of six concentrations (0.0006, 0.006, 0.06, 0.6, 6 and 60 mg/mL) were assayed in the different in vivo and in vitro assays, with the ADI being 0.06 mg/mL.

### 2.2. In Vivo Assays

#### 2.2.1. *Drosophila melanogaster*

*D. melanogaster* is a model organism whose results may be compared and extrapolated to mammals including humans [40].

The genetic background used in the present study consisted of two strains of *D. melanogaster*, each carrying one or more mutations on chromosome 3, affecting the phenotype of the wing and/or hairs and used as markers. The genetic background on chromosome 3 of these strains is as follows:

- *mwh*/*mwh*: (*multiple wing hairs*) carrying the recessive mutation *mwh* (multiple wing hairs) that, in homozygosis, produces multiple trichomes instead of one per cell [41].
- *flr^3^*/*ln (3LR)*, *TM3*, *rip^p^sep bx^34e^e^s^Bd^S^ (flare)* where *flr^3^* is a lethal recessive marker in homozygosis that produces deformed trichomes but is viable in homozygous somatic cells once larvae start their development [42]. The *Bd^S^* mutation is viable only in homozygosis. The closely linked lethal recessive persists on many of the chromosomes carrying the *Bd^S^* allele, so this allele is lethal in homozygosis and serves as a marker for the *flr^3^* mutation [43]. TM3 is a multiple inversion that prevents natural overcrossing in the area in which it is located. The TM3 inversion includes paracentric inversions on both arms of the chromosome as well as a pericentric inversion.

#### 2.2.2. Toxicity and Antitoxicity Assays

Toxicity and antitoxicity assays in *Drosophila melanogaster* were carried out following the protocol established by Graf, Würgler, Katz, Frei, Juon, Hall and Kale [44]. After crossing the two parental strains, groups of 100 individuals of 72-h-old larvae were transferred to tubes and were fed on a chronic treatment with the different concentrations of monosodium glutamate. 

The antitoxicity tests consisted of combined treatments of the same concentrations as in the toxicity assays, adding the oxidative toxin H_2_O_2_ at 0.12 M (Sigma-Aldrich, Darmstadt, Germany) [45]. Negative (H_2_O) and positive toxicant (0.12 M H_2_O_2_) concurrent controls were also assayed, according to previous ranges established by Romero-Jiménez et al. [46].

Three independent experiments were carried out for each trial at the different concentrations, and after a period of 10–12 days the percentage of flies emerged was evaluated with respect to the concurrent control according to the formula [(nº of individuals hatched in each treatment/nº of individuals hatched in the concurrent control) × 100], where n is the number of hatched flies. The standard deviation for the three experiments was also determined and the fitting graphic provided by the Microsoft Office Excel 2007 program was added. For statistical analysis, the chi-square statistical values were compared (*p* < 0.05) for the different concentrations analysed with respect to the negative control in the toxicity test; meanwhile, the statistical analysis of antitoxicity was carried out by comparing the values of the different concentrations with respect to the value of the positive control [47].

#### 2.2.3. Genotoxicity and Antigenotoxicity Assays (SMART)

The wing somatic mutation and recombination assay (SMART) uses two markers that affect the phenotype of wing cells: *mwh* and *flr^3^*. It has demonstrated accuracy in detecting point mutations, deletions, certain types of chromosomal aberrations and mitotic recombination [44]. Detectable recombinogenic activity in the SMART wing assay is quantifiable by analysing the wings of heterozygous *mwh*/TM3, *Bd^s^* flies. In these flies, multiple TM3 inversions prevent recombination processes in the affected region and, therefore, the detected spots are produced exclusively by mutation [48,49,50].

Genotoxicity assays were carried out following the procedure of Graf, Würgler, Katz, Frei, Juon, Hall and Kale [44], where groups of 100 trans heterozygous larvae were chronically fed with the ADI concentration of monosodium glutamate.

Antigenotoxicity tests were performed following the method described by Graf et al. [51], which consisted of combined treatments of genotoxin (H_2_O_2_ 0.12 M) and the same concentration used in the genotoxicity tests. After emergence, trans heterozygous *mwh/flr^3^* wings were mounted for mutation screening.

#### 2.2.4. Types of Spots or Clones

**Figure 1:**
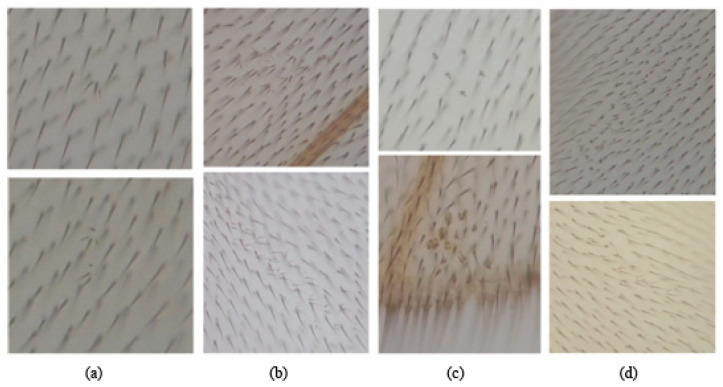
Representative homemade photographs were taken showing the different types of spots scored in the treated individual: (**a**) small *mwh* spots; (**b**) large *mwh* spots; (**c**) *flr^3^* spots and (**d**) twin spots.

The different types of spots that can be found under a photonic microscope in the SMART assay are classified according to the type of mutation and size induced by the tested substance in *Drosophila* cells [44], as detailed below (see Figure 1 for further details):

- Single *mwh* spots, formed by cells with 3 or more hairs each. The genetic alterations that can give rise to the *mwh* phenotype are point mutation, deletion, mitotic recombination between the *mwh* and *flr* loci, or no disjunction.
- Single *flr^3^* spots formed by cells of the flare phenotype. This phenotype is mainly manifested by a point mutation or deletion in the wild-type allele for this locus.
- Twin spots, formed by adjacent cells of the *mwh* and *flr^3^* phenotypes. They originate exclusively by mitotic recombination between the *flr* locus and the centromere.

Large spots have an elongated shape, longitudinal to the wing axis, indicating the main direction of growth [52], and are generally continuous. Sometimes, clusters of wild-type cells appear among the mutant cells, possibly due to a separation of the cells of a developing clone by internal tissue pressures or movements of independent cells. 

Twin spots and very large single spots are very rare in untreated fly wings and are caused by spontaneous mutations in the *Drosophila* embryo.

The wing of *Drosophila melanogaster* is composed of two cell layers, dorsal and ventral, and a total of 24,400 monotrichomes per wing [53]. In wild-type flies, each of these cells emits a single hair or trichome.

Wing analysis was performed under a microscope at 400× magnification and consisted of locating clones or individual cells showing the *mwh* or *flr^3^* mutant phenotype against a background of wild-type cells [44].

The evaluation of the data was carried out according to the multiple decision process described by Frei and Würgler [48,54]. In it, the frequencies of each type of mutant clone per wing were compared with their corresponding negative controls using the binomial test of Kastenbaum and Bowman [55], without Bonferroni correction and with a confidence level of 5%. Inconclusive and positive results are further analysed with the nonparametric Mann–Whitney U-test and Wilcoxon test (α = β = 0.05), which considers the range of values in controls and treatments [56].

The inhibition percentages (IP) for the combined treatments were calculated from the total number of spots per wing, with the following formula [57]:

IP = [(genotoxin − combined treatment)/genotoxin] × 100

#### 2.2.5. Longevity Assays

The longevity tests were carried out according to the procedure of Tasset-Cuevas et al. [58]. This assay was used to test the lifespan and quality of life provided by the compound under study to the model organism *Drosophila melanogaster*. After larvae were obtained, they were subjected to their specific treatment. After 12 days, 25 female and 25 male adults were placed in independent sterile special longevity tubes with double openings. Medium changes with the corresponding dilution and compound were carried out twice a week. At each change, the number of adults remaining alive and the number of casualties were recorded.

The statistical treatment of the survival data for each concentration tested was evaluated with the SPSS 17.0 statistical program (SPSS, Inc., Chicago, IL, USA), using the Kaplan–Meier method. The significance of the curves was determined using Mantel–Cox analysis (log-rank method) (*p* < 0.05).

### 2.3. In Vitro Assays

#### 2.3.1. HL-60 Cells

The possible in vitro antitumour activity of monosodium glutamate was evaluated in the promyelocytic cell line of human leukaemia HL-60, which was provided by Dr. José M. Villalba Montoro of the Department of Cell Biology of the Science Faculty of the University of Córdoba. In culture, HL-60 cells are ovoid or round, have large round nuclei, which are sometimes binucleate with distinct margins, fine chromatin and two to four nucleoli. The cytoplasm is basophilic with multiple prominent azurophilic granules [36].

#### 2.3.2. General Procedures

Cells were maintained in in vitro culture bottles with RPMI-1640 (Cat No. BE12-167F) (Cambrex Bio Science, BioWhittaker, Verviers, Belgium) liquid medium in an incubator (ShelLab, Cornelius, OR, USA) at 37 °C, with a humidified atmosphere, 5% CO_2_ and in dark conditions [33].

For the cell counting process, cells were stained with 10 μL of trypan blue (Fluka, CAT No. 93595) (Merck KGaG, Darmstadt, Germany), an exclusion dye used in cell viability counts (live cells exclude it and dead cells do not). Subsequently, cells were counted under an inverted optical microscope AE30/31 (MoticEurope S.L.U., Cabrera de Mar, Barcelona, Spain). The cell concentration, expressed as number of cells/mL, was obtained by multiplying the following:

Cells/mL of culture = (nº cells in chamber/4) × 2 × 10^4^

#### 2.3.3. Cytotoxicity Assay

To carry out the cytotoxicity assays, a multiwell plate with 96 wells was used, into which a final volume of 200 µL of the different concentrations of monosodium glutamate together with the corresponding cell volume containing 20,000 cells was deposited. A positive control was also established with RPMI medium standardised in our laboratory [59]. 

To study the cytotoxicity of monosodium glutamate, the trypan blue exclusion test was used. The results obtained were plotted graphically, using the means of three independent experiments of the viable cells. 

The relative growth curve and the standard deviation for the three experiments were determined and the fitting graphic was provided by the Microsoft Office Excel 2007 program.

#### 2.3.4. Internucleosomal DNA Fragmentation Assay

The main mechanism of cancer suppression is apoptosis or programmed cell death. One of the mechanisms by which it produces the cytotoxic effect in HL-60 cells undergoing treatment, and which is an indicator marker of apoptosis, is the detection of internucleosomal DNA fragmentation [60]. The fragmentation test was carried out following the method developed by Merinas-Amo, Merinas-Amo, Valenzuela-Gómez, Haro Bailón, Lozano, Mateo-Fernández, Fernández-Bedmar and Alonso Moraga [59]: 

A total of 10^6^ cells/mL were incubated in 12-well culture plates (Thermo Fisher Scientific Inc., Barcelona, Spain) and treated with monosodium glutamate at different concentrations or with RPMI only (control sample) during 5 h in the cell culture chamber. Subsequently, steps of lysis, precipitation, washing and hydration were carried out to isolate the DNA of treated HL-60 cells.

Samples were quantified in a spectrophotometer Nanodrop ND-1000, and 1200 ng of DNA stained with Green Go Taq (Nº CAT 0000224650) (Promega Biotech Ibérica S.L., Madrid, Spain), and including an internal lane size control was loaded on agarose gel (Condalab, Madrid, Spain) at 2%. Finally, electrophoresis (80 mA for 35 min) and gel development under ultraviolet light was performed due to the GelRed intercalated in our DNA that was included in the gel.

#### 2.3.5. Comet Assay

Induction of DNA damage at the single- and/or double-strand level was carried out using the alkaline comet assay (pH < 13). This experiment was performed based on the following methods previously described by Husseini et al. [61], Kumaravel et al. [62] and Prosperini et al. [63], with some modifications developed by Mateo-Fernández et al. [64]: 

HL-60 cells at a density of 106 cells/mL were resuspended in 1.5 mL of the ADI dilutions of monosodium glutamate during 5 h. Then, samples were washed and mixed in agarose gel. Subsequently, treated cells undergo alkaline lysis, electrophoresis and neutralisation steps to isolate the DNA. 

DNA from 50–100 cells was observed by treating the slides with 7 µL of propidium iodide (Sigma-Aldrich, P4170) for 5 min, and the emitted fluorescence was analysed with a fluorescence microscope Leica DM2500 at 400× (Leica Microsystems GmbH, Wetzlar, Germany) with a green filter and attached to a camera (JAI CV-M4CL). 

The *OpenComet* version of the *ImageJ* program (NIH) was used for cell damage analysis. To analyse the tail size (TM) caused by monosodium glutamate on the DNA integrity of HL-60 cells, we used the statistical program SPSS 17.0. Data were analysed by applying a one-way ANOVA and Tukey’s post hoc test (*p* < 0.05).

#### 2.3.6. Methylation Status Assay

DNA methylation at the 5′ position of cytosine residues within CpG dinucleotides by the addition of a methyl group is the best-known epigenetic mark [65]. DNA hypermethylation in the promoter region of many genes has been shown to be responsible for the silencing of more than 600 cancer-related genes [66,67,68]. On the other hand, it is known that global DNA hypomethylation activates endoparasitic sequences and causes general chromosomal instability, leading to various mutations and cancer progression [69].

The methylation status assay was carried out following the method developed by Merinas-Amo, Merinas-Amo and Alonso-Moraga [35]:

In the first step, from a volume corresponding to 500 ng of DNA isolated from HL-60 cells previously treated with the ADI concentration of monosodium glutamate, processes of DNA denaturation and bisulphite conversion were carried out with the commercial kit EZ DNA Methylation-Gold™ (Zymo Research, Irvine, CA, USA).

In the second step, a quantitative methylation-specific PCR (qMSP) was performed, in which the bisulphite-modified DNA was used and a quantitative real-time PCR based on specific fluorescence (qMSP) was performed on a MiniOpticon Real-Time PCR System thermal cycler (MJ Mini Personal Thermal Cycler, Bio-Rad Laboratories, Inc., Hercules, CA, USA). Finally, results were analysed using Bio-Rad CFX Manager 3.1 software.

To analyse a large region of genomic DNA, different repetitive elements were selected. Alu and LINE sequences are interspersed throughout the genome, whereas satellite sequences are only interspersed in regions of the centromere [70,71,72,73]. Alu C4, Alu M1, LINE-1 and Sat-α sequences, obtained from Isogen Life Science, were used for the evaluation of the DNA methylation status of HL-60 cells treated with monosodium glutamate at ADI concentration [74]. Further information about the selected primers was placed in the Supplementary Material and is presented in Table S1.

Relative performance results were normalised against the Alu C4 maintenance sequence and the comparative C_T_ method of Nikolaidis et al. [75] and Liloglou et al. [76], as follows:

- The C_T_ of the target gene was normalised with respect to the reference gene (ΔC_T_).
- We compared ΔC_T_ of each sample from each experiment or control (ΔC_T,r_) with ΔC_T_ of the calibrator sample (ΔC_T,cb_), ΔΔC_T_.

The relative value of each sample was defined using the following formula:

2^−(ΔCT,r−ΔCT,cb)^ = 2^−ΔΔCT^

Each sample was analysed in triplicate using the ANOVA statistical test and Tukey’s post hoc test via SPSS 17.0 statistical software to evaluate the differences between the monosodium glutamate and the repetitive elements.

## 3. Results

### 3.1. Toxicity and Antitoxicity

**Figure 2:**
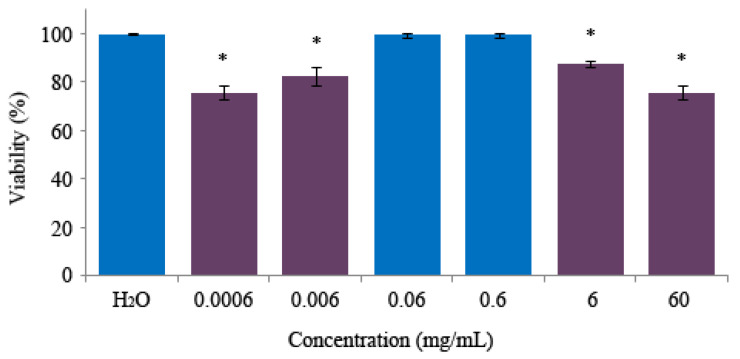
Toxicity levels of monosodium glutamate in *D. melanogaster*. Data are expressed as a percentage of surviving adults with respect to 300 untreated 72-hour-old larvae +/− SD from three independent experiments treated with different concentrations of monosodium glutamate. Asterisks (*) indicate significant differences (one tail) with respect to the control group. * Chi-square value higher than 5.02. The original results of the three experiments were placed in the Supplementary Materials and are presented in Table S2.

The relative percentages of emerging *D. melanogaster* after treating larvae with monosodium glutamate showed a non-significant result compared with the control at the two-medium concentrations tested (Figure 2). The ADI concentration (0.06 mg/mL) exhibited a non-toxic effect on the fly viability, resulting in100% survival. The other concentrations assayed presented a significant decrease in survival that ranged between 87 and 75.8% with respect to the control, although the considered toxic level, the lethal dose 50 (LD_50_), was not reached in any test carried out [64]. This confirms that the monosodium glutamate ADI concentration established by the JECFA is a safe concentration, as well as the validity of the assay [14,38], so the biological significance of these results agrees with different studies that confirm that monosodium glutamate is a safe additive.

**Figure 3:**
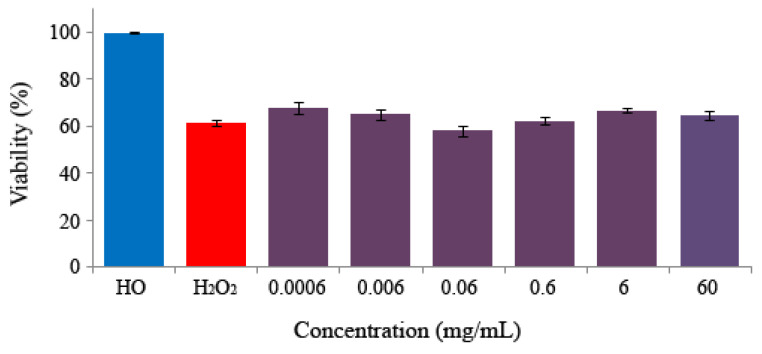
Antitoxicity levels of monosodium glutamate in *D. melanogaster*. Data are expressed as a percentage of surviving adults with respect to 300 untreated 72-hour-old larvae +/− SD from three independent experiments treated with different concentrations of monosodium glutamate combined with 0.12 M hydrogen peroxide. The original results of the three experiments were placed in the Supplementary Materials and are presented in Table S3.

Results of survival percentage of treated *Drosophila* in combination with 0.12 M H_2_O_2_ (Figure 3) indicate that monosodium glutamate does not have the ability to protect the flies against oxidative stress due to the viability percentages of treatments, showing a range similar to the positive control (between 57 and 67% for monosodium glutamate and 62% for control).

The lack of concordance about the effects of monosodium glutamate in *Drosophila* between the toxicity and antitoxicity assays could be due to each substance displaying antioxidant or prooxidant activities in a competitive way against the effects of hydrogen peroxide when both are combined [77].

A wide range of research affirms the effect that hydrogen peroxide has: it can interact directly with DNA or modulate transcription and suppress genomic repair pathways; induce microsatellite instability in germ cells of *D. melanogaster* [78]; produce genetic damage by its electrophilic compounds generated [79]; and it is well established that hydrogen peroxide is an endogenous mutagen responsible for some of the highest cancer risks associated with persistent inflammation [80]. Oxy radicals derived from hydrogen peroxide can act either directly on the genome, causing chromosome damage that induces oncogenic mutations [81,82], or indirectly by modulating gene transcription [83,84] or by suppressing genome repair pathways [85,86]. Moreover, a study of genotoxicity induced by hydrogen peroxide using the in vivo *Drosophila* assay [87] indicated that the oxidative agent can induce somatic mutation and mitotic recombination (concentration ranged between 0.12 M and 0.48 M). The relative contribution of the recombinational events to the total clone induction was estimated by comparing the frequency of *mwh* spots on the marker wings with the frequency of *mwh* spots in the balancer wings. In this respect, an average of 60% of the clones showed a recombinational origin.

### 3.2. Genotoxicity and Antigenotoxicity

Table 1 shows the results of genotoxicity and antigenotoxicity assays of monosodium glutamate at the ADI concentration using the *D. melanogaster* model. The total frequencies of clones per wings for negative and positive controls were 0.158 and 0.400, respectively. These data were ranged in a similar previous study that established the concentration for controls [46]. After applying the binomial Kastenbaum–Bowman Test, the inconclusive results for the entire simple treatment were identified. Next, the Mann–Whitney U-test revealed that there were no differences between the treatment and the concurrent control (0.263 clones per wings) (indicated by Delta marker in Table 1). To conclude, we highlight the non-genotoxic effect of monosodium glutamate due to no significant mutational effect shown in the SMART test at the ADI concentration.

Results about the antigenotoxicity assay when monosodium glutamate is combined with H_2_O_2_ at the ADI concentration were inconclusive for the clones per wings rate (0.225) and required Mann–Whitney U-test statistical analysis to confirm that this concentration was not like the corresponding positive control (0.400). Moreover, the calculated inhibition percentage (IP) showed a genomic protection capacity of about 43.75% with respect to the positive control.

### 3.3. Longevity

**Figure 4:**
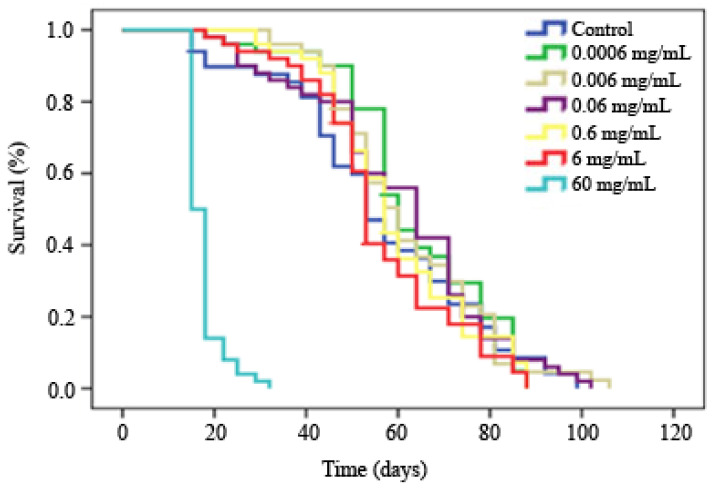
Survival curves of *D. melanogaster* fed with different concentrations of monosodium glutamate. A total of 25 female and 25 male individuals were used during the test by feeding them throughout all their life extension.

Results of the lifespan curves obtained by the Kaplan–Meier method showed no significant increase in *Drosophila* life extension at any concentration assayed of monosodium glutamate, except for the highest one (60 mg/mL), which significantly decreased the longevity of flies in 39 days with respect to the control (Figure 4 and Table 2). 

Regarding the health span results, all concentrations assayed significantly improved the quality of life of *Drosophila* over 11–16 days except for the highest concentration that decreased it in 12 days with respect to the control (Table 2). Sohet al. [88] argue that the quest for increased longevity must be accompanied by an increase in quality of life. This is quantifiable by studying the health span, which evaluates the survival curve when 75% of individuals are alive and which was also determined.

The positive data support the nutraceutical potential of monosodium glutamate, except for the highest concentration tested. The data provided to the research community by the present study could be related to the controversial results observed in the database of food additives [13,14]. Nevertheless, because of the lack of information about monosodium glutamate in similar experiments, it is important to deal with these results with caution and carry on with additional assays with other organisms.

### 3.4. Cytotoxicity

**Figure 5:**
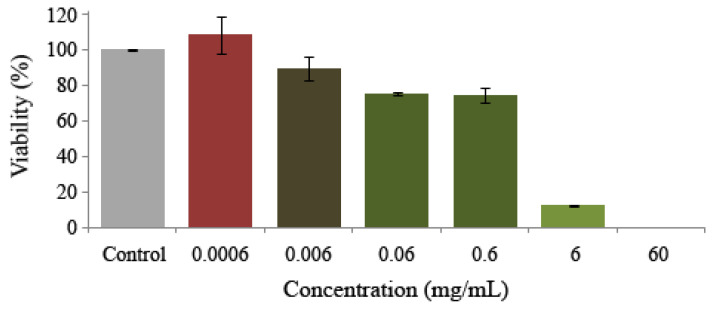
Effect of monosodium glutamate on cell viability. Viability in promyelocytic human leukaemia cells (HL-60) treated with different concentrations of monosodium glutamate for 72 h. Each point represents the growth percentage compared to the control, from an initial volume containing 20,000 cells at each treatment. Values are mean +/− SD from three independent experiments. The original results of the three experiments were placed in the Supplementary Materials and are presented in Table S4.

Results about viability of HL-60 cells treated with different concentrations of monosodium glutamate indicated a dose-dependent cytotoxic activity of the additive against the HL-60 cell line by inducing a decrease in tumour cell growth (Figure 5). The viability percentages ranged between 108 and 0% with respect to the control. Although the ADI concentration showed light cell growth inhibition at about 25% with respect to the control, the two highest concentrations tested (6 and 60 mg/mL) were able to reach the inhibitory concentration 50 (IC_50_), confirming its chemo preventive potential.

### 3.5. Internucleosomal DNA Fragmentation

**Figure 6:**
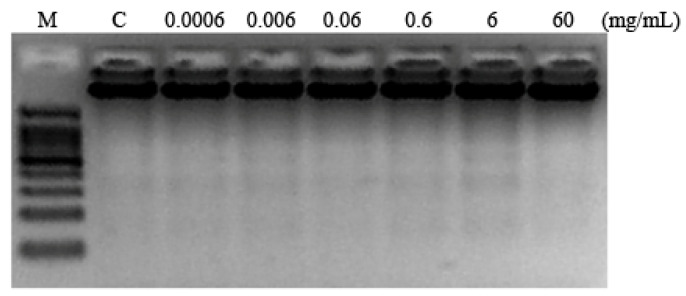
Internucleosomal DNA fragmentation. DNA-induced damage in promyelocytic HL-60 cells treated with different concentrations of monosodium glutamate for 5 h. M indicates DNA size marker; C indicates control treatment.

Internucleosomal DNA fragmentation is represented by DNA laddering, and it is associated with the activation of apoptosis in cancer cells, being a hallmark of the genomic integrity [60,89]. A fragmentation effect is not clearly observed at any concentration with respect to the control, which means a non-activation of apoptosis. Only in the 6 mg/mL concentration is DNA laddering lightly observed, a fact indicating that it is related with the cytotoxicity results previously obtained (Figure 6).

### 3.6. Comet Assay

**Figure 7:**
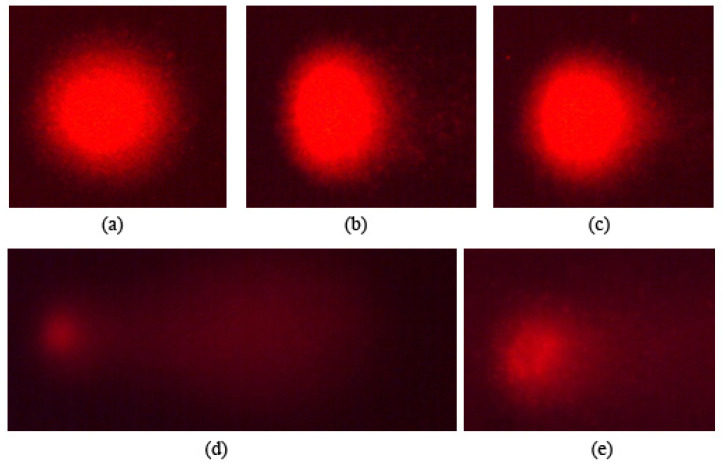
DNA strand break induction. DNA single- or double-strand break inductions in HL-60 cells treated with monosodium glutamate for 5 h. DNA migrations are reported as mean TM. (**a**) Negative control: untreated cells; (**b**,**c**) alkaline comet assay (pH < 13) of HL-60 cells treated with monosodium glutamate 0.06 mg/mL; (**d**,**e**) positive control: treated cells with a compound that induces DNA damage [89].

The ability of the compounds to induce strand breaks in the DNA structure was determined by the alkaline comet assay. Figure 7 shows representative images of the comet assay for monosodium glutamate at the ADI concentration (0.06 mg/mL). Following the method described by Almeida-Lima et al. [90] and Fabiani et al. [91], we quantitatively evaluated the genetic damage induced by our additive in HL-60 cells by visually assigning numbers from 0 to 4 (0 being a cell without damage and 4 being a very damaged cell) directly to each of the obtained cells. Subsequently, a statistical treatment was carried out using the non-parametric Mann–Whitney U-test. All our results were between the values of 0 and 1, which indicates that no significant genomic damage was induced at the single- or double-strand level of the DNA of human leukaemia cells treated at the established ADI concentration.

The comet assay makes it possible to identify the morphological damage caused at the DNA level by our compounds at the single-cell level [92]. According to Fairbairn and O’Neill [93], TM measurements in the comet assay are related to those obtained in cytotoxicity. Our results agree with the mentioned correlations, since the TM values are associated with the percentage of cell viability obtained by the trypan blue exclusion test in HL-60 cells: at its ADI concentration, monosodium glutamate does not induce cell damage, and its percentage of cell viability is between 75% with respect to the control. In addition, the results of DNA fragmentation agree with those obtained in the comet, since TM values < 1 indicate that no DNA damage has occurred [91].

### 3.7. Methylation Status

**Figure 8:**
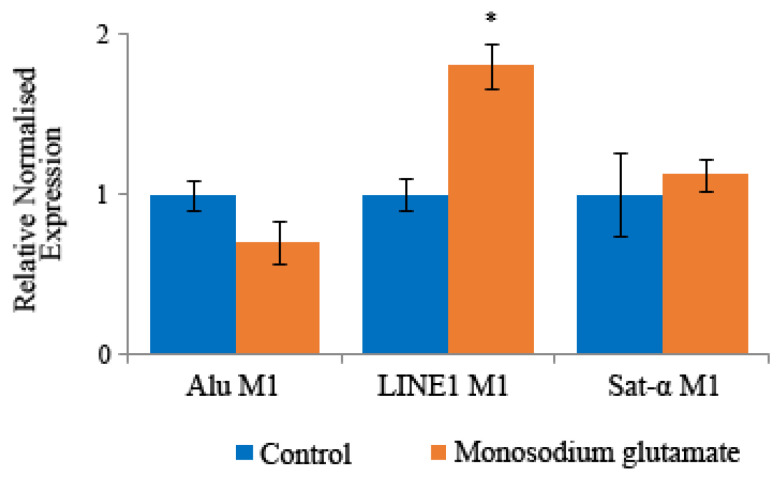
Methylation status. Relative normalised expression data of each repetitive element in HL-60 cells with monosodium glutamate (0.06 mg/mL). * Indicates significant difference value compared to the negative control by applying the one-way ANOVA test and post hoc Tukey’s test.

The normalised relative expression of the three repetitive sequences studied (Alu M1, LINE1 M1 and Sat-α) were studied after treatment of HL-60 cells with monosodium glutamate, conversion to bisulphite-modified DNA and qMSP steps (Figure 8).

After the analysis of the results with the ANOVA and posthoc Tukey tests, significant changes were observed in the methylation pattern of the tumour cells: the treatment of human leukaemia cells with monosodium glutamate produced a significant increase in the methylation of the LINE1 M1 sequences, regarding our control.

Considering that the methylation of repetitive sequences is considered as a genomic protection mechanism [23,94], monosodium glutamate can be considered as an interesting chemo preventive compound, since it can inhibit the effects of tumour cells totally or partially, in some of the sequences and for the concentration studied.

## 4. Discussion

Worldwide, monosodium glutamate is utilized as a flavour enhancer and is composed of an essential amino acid for nutrition. Despite visible improvements in legislation and the production of safer additives, many issues remain unresolved, leading to growing controversy and constant demand for improved food additives.

Flavours enhancers are used to complement or enhance the flavour of foods, but do not contribute their own flavour. One of the most widely used flavour enhancers is monosodium glutamate. Monosodium glutamate is a salt of glutamic acid, a natural amino acid. It is used in foods to enhance their natural flavour and to produce the umami flavour (pleasant salty taste), either alone or in synergy with disodium inosinate or disodium guanylate. In 2000, a review of previous safety assessments by the FAO, WHO and the Scientific Committee on Food for monosodium glutamate concluded that, although a part of the population is sensitive, this additive could be consumed without any concern [95]. Recently, other detrimental effects have been attributed to monosodium glutamate in murine and human models, since there are studies that confirm the induction of lipid peroxidation, the deterioration of synaptic plasticity of mice neurons, the harmful effects to murine oocytes and the increased development of overweightness in Chinese adults who consume this additive [96,97,98]. However, other scientists found no correlation between monosodium glutamate and obesity in the Chinese population and claim that supplementation of pig feed with this additive is safe and improves growth performance [99,100].

Our in vivo results are related to the favourable effects previously obtained by other researchers, since no significant detrimental effects are observed in the different studies of toxicity, antitoxicity, genotoxicity, antigenotoxicity and longevity. Moreover, a significant improvement in health span in treated *Drosophila* was observed at the different concentrations tested with monosodium glutamate, except for the highest concentration. In terms of safety, monosodium glutamate consumption is thought to be associated with various diet-related diseases such as obesity, diabetes, metabolic disorders and carcinogenesis [16,17,18,19]. However, monosodium glutamate is one of the most intensely studied food ingredients in the food supply and has been found safe; the Joint Expert Committee on Food Additives of the United Nations Food and Agriculture Organization and World Health Organization placed it in the safest category for food additives [101]. In relation to the effects of monosodium glutamate on life at the individual level, no previous results have been reported for the longevity and healthspan assays. In order to know the quality of life of the *Drosophila* treated in the longevity assays, we studied the top 25% of individual survival of the lifespan curves obtained in the previous test for each concentration tested. This part of the lifespan is considered as the healthspan of a curve, characterized by low and more or less constant age-specific mortality rate values [88]. 

The in vitro results show a dose-dependent cytotoxic effect for monosodium glutamate treatments with HL-60 tumour cells and a genomic protective effect of said additive in repetitive LINE sequences. Carcinogenesis is another argued notable side-effect of chronic monosodium glutamate consumption [102]. In addition to the production of ROS, monosodium glutamate can also lead to increased micronucleus frequency and chromosomal abnormalities [103]. The undesirable effects of monosodium glutamate are considered valid biomarkers of carcinogenesis among experts [103]. The cytotoxic pattern observed in the lower concentration of monosodium glutamate was also induced by folic acid in a similar test condition [89], where the lack of cytotoxic activity of folic acid suggests that it could have a promoter effect for cancer in humans. The meta-analysis published by Wien et al. [104] using 10 randomised controlled trials showed a borderline significant increase in frequency of overall cancer in the folic acid group compared to controls. Furthermore, monosodium glutamate-induced DNA damage includes single- and double-stranded breaks, mutations, clumping and stickiness of chromosomes, genetic rearrangements, impaired excision repair machinery and structural and numerical chromosomal aberrations [102,103]. The latter is considered a key biomarker for assessing cancer risk [103]. Several studies found that monosodium glutamate consumption is related to increased chromosomal aberration frequencies in human cell cultures, suggesting that monosodium glutamate has genotoxic and cancerous effects on the host cell genome [19,102,103,105].

Analysing all our results from a quantitative point of view for the different assays and additive, we only observed beneficial effects for the *Drosophila* quality of life assay and a neutral effect for the genotoxicity, antigenotoxicity and longevity assays (atthe ADI concentration) in said model organism in vivo. However, for the rest of the tests, the effect produced by our studied additive is detrimental both at the individual and cellular level.

These controversial data with respect to the current known data supporting the safe consumption of additives may be due to the variety of conditions used: possible differential responses of the organism used against each substance and by the biological level in which it was acting; type of cell line used; in vitro and in vivo conditions; concentration range; or even the test conditions.

Due to the high consumption that the different food additives have today, and the wide variety of results obtained, it is necessary to continue carrying out scientific studies and strict reviews by the EFSA to confirm a safe dose and use for each additive.

## 5. Conclusions

Diet is one of the most important environmental agents that exerts a significant effect on health and consequently on disease risk. The biological effects that monosodium glutamate showed on animal and cellular model systems have been studied in multilevel assays (molecular, cellular, individuals and populations), adding a new data corpus to science in the nutraceutical area.

Based on the results obtained in the different in vivo and in vitro tests on its safety, nutraceutical potential and chemo preventive potential, monosodium glutamate shows a safe character at individual and genomic levels and an improvement in quality of life in the in vivo tests. Moreover, it shows slight cytotoxic and DNA damage activity in tumour cells.

We can highlight the slight or non-significant positive nutraceutical and chemo preventive potential that monosodium glutamate shows at the ADI concentration as a safe dose for moderate consumption. 

For all the above mentioned, in addition for making responsible use and consumption of foods that contain monosodium glutamate and continuing with the periodic evaluations by the different food committees, more studies are also needed that allow us to elucidate the biological activities and the nutraceutical potential of the most clearly studied food additive.

## Figures and Tables

**Table 1:** Genotoxicity and antigenotoxicity of monosodium glutamate (0.06 mg/mL) in *Drosophila* wing spot test.

	Clones per Wings (Number of Spots) ^(1)^						
Compound	Nº Wings	Small Single Spots (1–2 Cells) m = 2	Large Single Spots (>2 Cells) m = 5	Twin Spots m = 5	Total Spots m = 2	Mann–Whitney Test ^(2)^	IP ^(3)^
H_2_O	38	0.105 (4)	0.053 (2)	0	0.158 (6)		
H_2_O_2_	40	0.200 (8)	0.200 (8)	0	0.400 (16) +		
Genotoxicity	38	0.158 (6)	0.105 (4)	0	0.263 (10) i	Δ	
Antigenotoxicity	40	0.125 (5)	0.100 (4)	0	0.225 (9) i	Δ	43.75

^(1)^ Statistical diagnosis according to Frei and Würgler [48]. + (positive), i (inconclusive) versus negative (H_2_O) or positive control (H_2_O_2_). m: multiplication factor. Kastenbaum–Bowman Test without Bonferroni correction, probability levels α = β = 0.05. ^(2)^ Mann–Whitney test was used when appropriate to resolve inconclusive results. Delta symbol (∆) means that there are not significant differences with respect to the concurrent control. ^(3)^ Inhibition percentage value for the combined treatment was calculated from total spots per wing according to Abraham [57].

**Table 2:** Mean and significance data of lifespan and health span.

Concentration (mg/mL)	Mean Lifespan (Days)		Mean Health Span (Days)	
Control	56.424		27.857	
0.0006	63.182	ns	43.769	*
0.006	61.950	ns	42.583	*
0.06	60.640	ns	41.833	*
0.6	59.380	ns	40.917	*
6	55.971	ns	36.250	*
60	17.672	*	15.000	*

Means were calculated by the Kaplan–Meier method and significance of the curves was determined by the log-rank method (Mantel–Cox). ns: non-significant (*p* > 0.05); *: significant (*p* < 0.05).

## Data Availability

The original contributions presented in this study are included in the article/Supplementary Material; further inquiries can be directed to the corresponding author.

## Supplementary Materials

The following supporting information can be downloaded at: https://www.mdpi.com/article/10.3390/foods13233981/s1, Table S1. Detailed primer information; Table S2. Original data of emerging adults obtained after chronically feeding *Drosophila* from three independent toxicity experiments: Table S3. Original data of emerged adults obtained after chronically feeding *Drosophila* from three independent antitoxicity experiments: Table S4. Original data of leukemia cells growth treated with monosodium glutamate during 72 h from three independent experiments.
